# The targeted anti‐oxidant MitoQ causes mitochondrial swelling and depolarization in kidney tissue

**DOI:** 10.14814/phy2.13667

**Published:** 2018-04-02

**Authors:** Esther M. Gottwald, Michael Duss, Milica Bugarski, Dominik Haenni, Claus D. Schuh, Ehud M. Landau, Andrew M. Hall

**Affiliations:** ^1^ Institute of Anatomy University of Zurich Zurich Switzerland; ^2^ Department of Chemistry University of Zurich Zurich Switzerland; ^3^ Center for Microscopy and Image Analysis University of Zurich Zurich Switzerland; ^4^ Department of Nephrology University Hospital Zurich Zurich Switzerland

**Keywords:** Anti‐oxidant, kidney, mitochondria, MitoQ

## Abstract

Kidney proximal tubules (PTs) contain a high density of mitochondria, which are required to generate ATP to power solute transport. Mitochondrial dysfunction is implicated in the pathogenesis of numerous kidney diseases. Damaged mitochondria are thought to produce excess reactive oxygen species (ROS), which can lead to oxidative stress and activation of cell death pathways. MitoQ is a mitochondrial targeted anti‐oxidant that has shown promise in preclinical models of renal diseases. However, recent studies in nonkidney cells have suggested that MitoQ might also have adverse effects. Here, using a live imaging approach, and both in vitro and ex vivo models, we show that MitoQ induces rapid swelling and depolarization of mitochondria in PT cells, but these effects were not observed with SS‐31, another targeted anti‐oxidant. MitoQ consists of a lipophilic cation (Tetraphenylphosphonium [TPP]) joined to an anti‐oxidant component (quinone) by a 10‐carbon alkyl chain, which is thought to insert into the inner mitochondrial membrane (IMM). We found that mitochondrial swelling and depolarization was also induced by dodecyltriphenylphosphomium (DTPP), which consists of TPP and the alkyl chain, but not by TPP alone. Surprisingly, MitoQ‐induced mitochondrial swelling occurred in the absence of a decrease in oxygen consumption rate. We also found that DTPP directly increased the permeability of artificial liposomes with a cardiolipin content similar to that of the IMM. In summary, MitoQ causes mitochondrial swelling and depolarization in PT cells by a mechanism unrelated to anti‐oxidant activity, most likely because of increased IMM permeability due to insertion of the alkyl chain.

## Introduction

Kidneys are highly aerobic organs and receive 20–25% of cardiac output. Renal proximal tubules (PTs) are densely packed with mitochondria, which are required to produce ATP to power processes like solute transport. Kidney mitochondria can be damaged by various insults – including ischemia, sepsis and toxins – and mitochondrial dysfunction is implicated in the pathogeneses of numerous renal diseases (Che et al. 2014; Ralto and Parikh 2016; Bhargava and Schnellmann 2017). Mitochondria are thought to produce reactive oxygen species (ROS) as a by‐product of oxidative phosphorylation (OXPHOS) (Murphy 2009), and increased ROS generation in damaged mitochondria can lead to oxidative stress and activation of cell death pathways (Halestrap 2010). Lowering mitochondrial production or quenching ROS in disease states might therefore be protective for cells. Since non‐specific anti‐oxidants have proven disappointing (Cocheme and Murphy 2010), and non‐mitochondrial ROS can have important physiological roles (Sedeek et al. 2013), several anti‐oxidant compounds have been developed that specifically target mitochondria, including SS peptides, plastoquinone analogues (SkQ1/SkQR1), and MitoQ (Tabara et al. 2014; Hall and Schuh 2016). SS peptides, such as SS‐31, are thought to interact with cardiolipin in the inner mitochondrial membrane (IMM) (Szeto and Birk 2014), whilst plastoquinone analogues and MitoQ contain lipophilic cations and utilize the voltage gradient across the IMM (Δ*ψ*_m_) generated by the OXPHOS complexes to selectively accumulate into the mitochondrial matrix at very high concentrations (Smith and Murphy 2011; Jankauskas et al. 2012).

The lipophilic cation in MitoQ (Tetraphenylphosphonium [TPP]) is joined to an anti‐oxidant component (quinone) by a 10‐carbon alkyl chain, which is thought to insert into the IMM (Smith and Murphy 2010). Previous studies have shown that MitoQ accumulates into mitochondria up to 100–1000 fold, and uptake into kidney tissue has been confirmed in vivo (Smith and Murphy 2010). Numerous recent animal studies have suggested beneficial effects of MitoQ in various different renal diseases, including: acute kidney injury (AKI) induced by ischemia‐reperfusion injury (IRI) (Dare et al. 2015), cold storage (Parajuli et al. 2012), sepsis (Lowes et al. 2008) and cisplatin (Mukhopadhyay et al. 2012); diabetic nephropathy (Chacko et al. 2010; Xiao et al. 2017); polycystic disease (Ishimoto et al. 2017); and cystinosis (Galarreta et al. 2015). Due to the promising protective effects in preclinical studies, MitoQ is now being trialed in humans (Smith and Murphy 2010). However, since MitoQ accumulates into mitochondria at very high concentrations, the possibility remains that it could have effects on mitochondrial function other than on ROS levels, not all of which might be beneficial. For example, a recent study has reported that MitoQ can actually *increase* ROS production in some cancer cells, and this is associated with a decrease in Δ*ψ*_m_ and mitochondrial DNA (mtDNA) copy number (Pokrzywinski et al. 2016). Moreover, MitoQ has also been found to induce autophagy in liver cells (Sun et al. 2017). Thus, there is a pressing need to better understand the detailed effects of MitoQ on mitochondrial function in kidney tissue.

Mitochondria are complex and dynamic organelles, capable of showing rapid changes in morphology (Archer 2013). Live cell fluorescence imaging allows the study of both the structure *and* function of mitochondria, in their native environment within living tissue (Duchen et al. 2003). Using this approach, we have made the surprising discovery that MitoQ causes rapid swelling and depolarization of mitochondria in kidney PT cells, and that this effect is unrelated to its intended anti‐oxidant properties. Therefore, caution should be exercised before transitioning this treatment to humans with renal disease.

## Materials and Methods

Unless stated otherwise, all reagents were purchased from Sigma Aldrich. MitoQ was acquired from MedKoo Biosciences and SS‐31 from China Peptides.

### Cell lines

Opossum kidney (OK) cells were a kind gift from the group of Prof O. Devuyst (Physiology, University of Zurich).

### Animals

All experiments were performed on 8‐ to 12‐week‐old male C57BL/6JRj mice.

### Live cell imaging

Cells were seeded on Poly‐L‐lysine coated coverglasses (Hecht‐Assistent) and grown to 90% confluence. Imaging was performed at 37°C in a buffer adjusted to pH 7.4 containing (in mmol/L): 138 NaCl, 5.6 KCl, 1.2 NaH_2_PO_4_, 2.6 CaCl_2_, 1.2 MgCl_2_, 10 Glucose, 4.2 NaHCO_3_, and 10 HEPES. Tetramethylrhodamine methyl ester (TMRM) was purchased from Thermo Scientific and was used at 50 nmol/L (excitation 555 nm). Confocal microscopy was performed on a Leica SP8 inverse STED 3X.

Kidney cortex tissue slices were generated from freshly externalized organs in mice anaesthetized with intra‐peritoneal ketamine (0.065 g/kg) and xylazine (0.01 g/kg), according to previously established protocols (Hall et al. 2009). After the removal of the capsule one pole of the kidney was mounted and cut with a vibratome (Microm HM 650 V, Thermo Scientific) into 250 *μ*m thick sections. The tissue was kept until usage at 4°C in a physiological buffer adjusted to pH 7.4 and gassed with carbogen (95% O_2_/5% CO_2_), containing (in mmol/L): 118 NaCl, 4.7 KCl, 1.2 KH_2_PO_4_, 1.8 CaCl_2_, 1.44 MgSO_4,_ 5 glucose, 10 NaHCO_3_, 10 HEPES, 5 sodium pyruvate, 2.5 sodium butyrate, and 2.5 sodium lactate. For live imaging slices were mounted in a heated chamber (Warner Instruments) containing oxygenated buffer. Imaging was performed with a Leica TCS SP8 Upright MP FLIM Microscope equipped with an HC IRAPO L 25x/1.0 W motCORR, WD: 2.6 mm and an Insight DS+ Dual (680–1300 nm & 1041 nm) ultrafast NIR laser for multiphoton excitation. Slices were incubated for 1 h before experiments in TMRM (50 nmol/L), which was excited at 850 nm.

### Oxygen consumption

Cells were plated in Seahorse XFp 8 well culture plates 1 day prior to experiments. 1 h before analysis cells were incubated at 37°C without CO_2_, in the same buffer used for live imaging experiments. Cartridges were filled with stock solutions of drugs dissolved in the buffer. After each injection oxygen consumption was measured in 3 cycles, each consisting of 2 min mixing followed by 2 min of measurement. Values were normalized to the basal rate.

### Liposomes

Liposomes were prepared according to a previously established protocol (Firsov et al. 2016). Excess Calcein was removed with dialysis (Slide‐A‐Lyzer MINI 7K, Thermo Fisher Scientific) at 4°C for 48 h against a buffer containing 100 mmol/L KCl, 10 mmol/L Tris, and 10 mmol/L MES, and adjusted to pH 7.4. The assay was performed in dialysis buffer. Fluorescence was recorded every minute at 528/20 nm (excitation 485/20 nm) using a Synergy 2 Multidetection reader (Biotek). Baseline was measured with liposomes only (500 ng/mL) for 5 min before addition of drugs.

### Study approval

All animal experiments were approved by the Zurich Cantonal Veterinary Office.

## Results

### MitoQ causes acute swelling and depolarization of mitochondria in kidney proximal tubule cells

**Figure 1:**
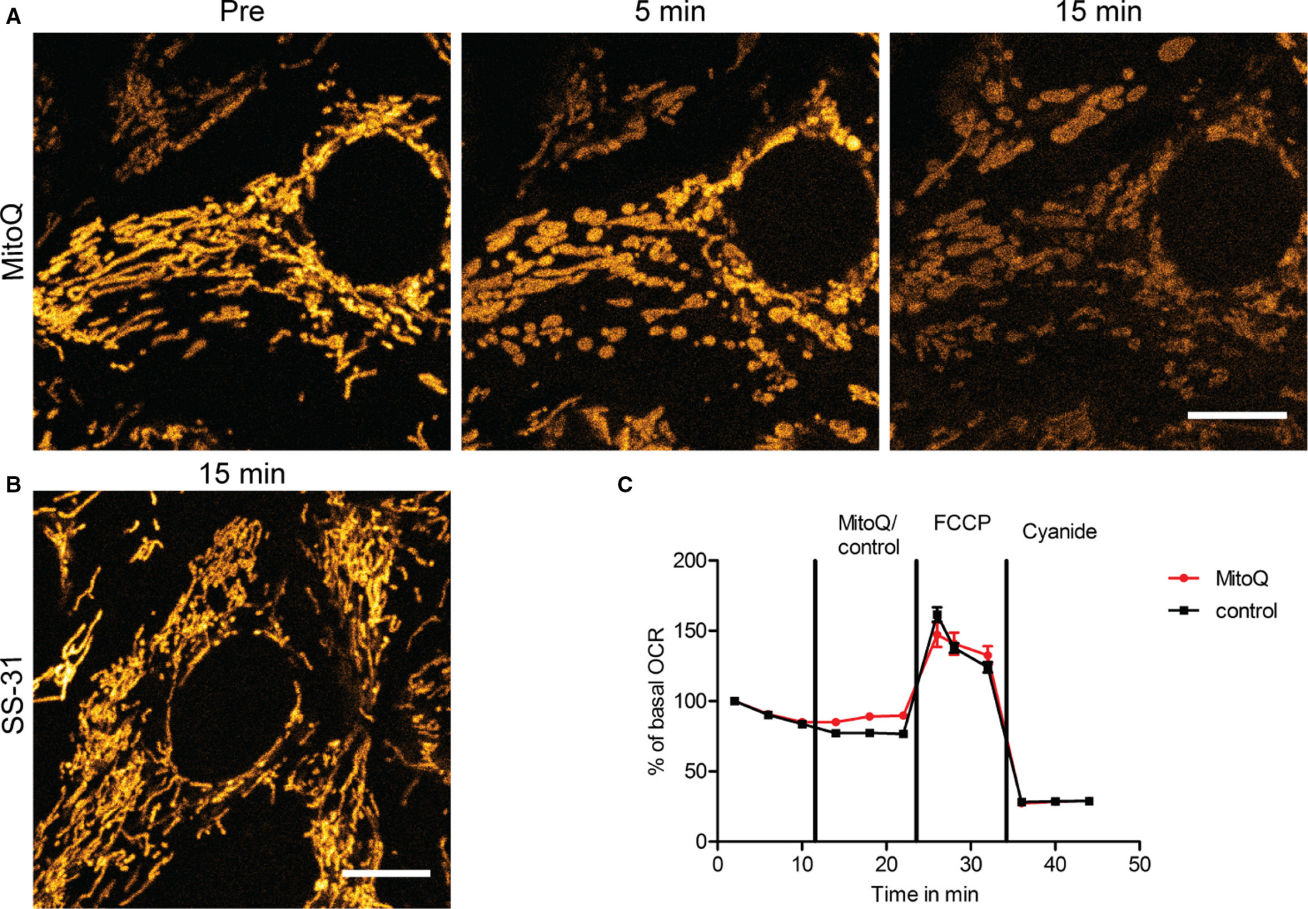
MitoQ induces mitochondrial swelling in proximal tubular cells. (A) Live confocal imaging of proximal tubule‐derived OK cells loaded with the membrane potential dependent dye TMRM revealed that mitochondria swelled rapidly in response to MitoQ (500 nmol/L) before subsequently depolarizing (scale = 10 *μ*m). (B) In contrast, the antioxidant SS‐31 at a 1000 fold higher dose (500 *μ*mol/L) did not induce changes in mitochondrial morphology or energization (scale = 10 *μ*m). (C) Addition of MitoQ (500 nmol/L) did not acutely decrease baseline oxygen consumption rate (OCR) in OK cells. Moreover, maximum OCR poststimulation of OXPHOS with the uncoupler FCCP (1 *μ*mol/L) was similar in both MitoQ and vehicle treated cells. OXPHOS was subsequently inhibited with cyanide (2 mmol/L). All values were normalized to baseline OCR (*n* = 3 experiments).

To investigate the effect of MitoQ on mitochondrial structure and function we performed live confocal imaging in PT‐derived OK cells, since these retain many important transport processes needed for normal kidney function (Eshbach et al. 2017). Cells were loaded with the voltage dependent dye TMRM to assess changes in Δ*ψ*_m_ in real time. Application of MitoQ (500 nmol/L) caused acute swelling of mitochondria (within 5 min) and rapid dissipation of Δ*ψ*_m_ (Fig. 1A). In contrast, SS‐31 at a 1000 fold higher dose (500 *μ*mol/L) had no discernible impact on mitochondrial structure/function (Fig. 1B), showing that the response to MitoQ is unique to this compound, rather than a common feature of all mitochondrial targeted anti‐oxidants.

### MitoQ does not acutely inhibit oxygen consumption

To explore whether MitoQ‐induced mitochondrial swelling is associated with changes in OXPHOS activity further experiments were performed to assess the effect of this compound on oxygen consumption rate (OCR). We found that the addition of MitoQ (500 nmol/L) did not acutely lower baseline OCR in OK cells, or change the maximum OCR achieved in response to stimulation of OXPHOS with the uncoupler Carbonyl cyanide‐4‐(trifluoromethoxy)phenylhydrazone (FCCP) compared to control cells (Fig. 1C). Thus, mitochondrial swelling and depolarization induced by MitoQ occurs in the absence of an acute decrease in OXPHOS function or capacity.

### Mitochondrial toxicity from MitoQ is not related to its anti‐oxidant properties

**Figure 2:**
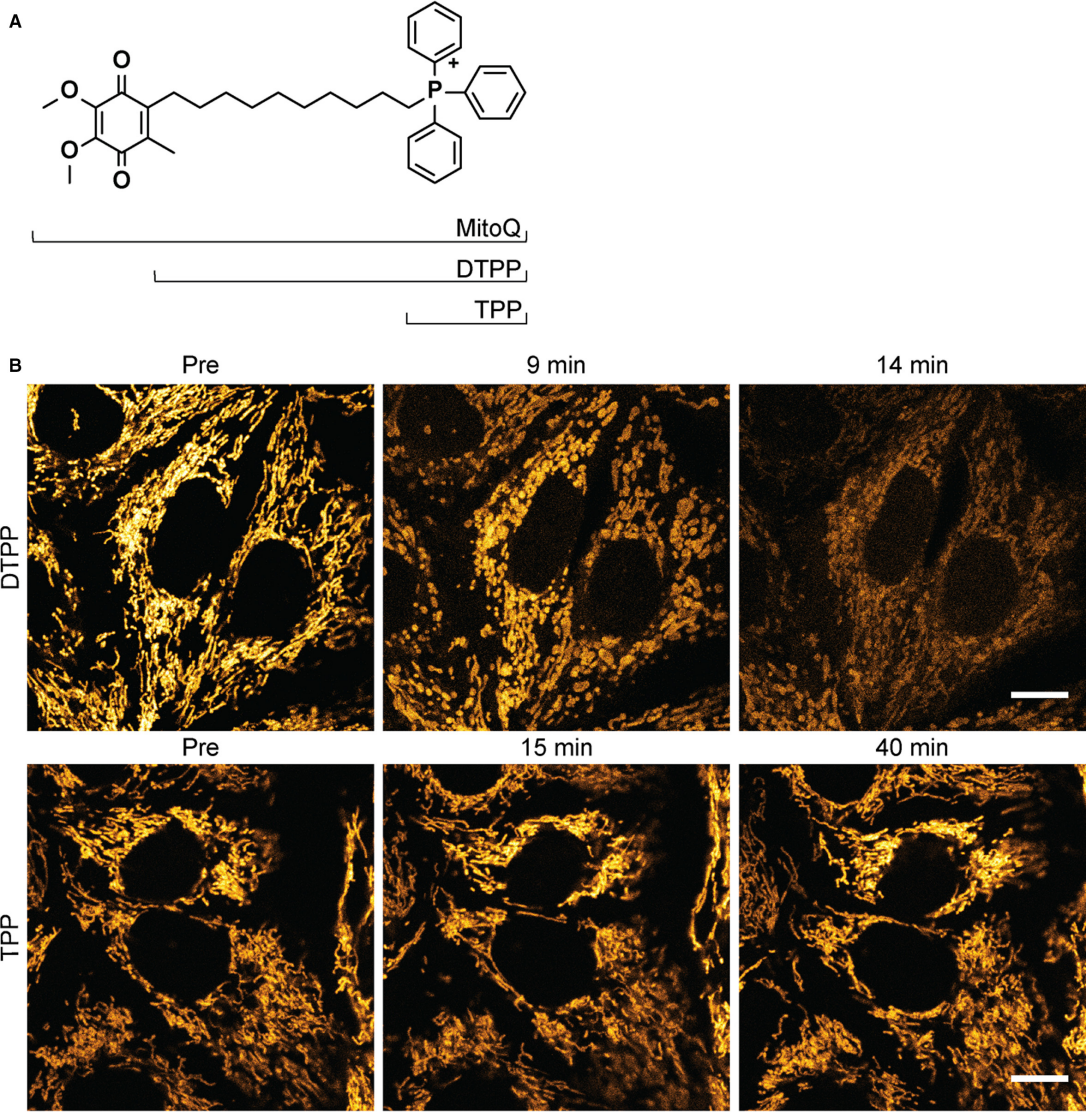
MitoQ‐induced mitochondrial swelling is independent of its anti‐oxidant function. (A) Chemical structures of MitoQ, DTPP and TPP. (B) Live confocal imaging of OK cells loaded with TMRM revealed that DTPP (500 nmol/L) induced mitochondrial swelling and depolarization, identical to that caused by MitoQ. In contrast, TPP (500 nmol/L), which lacks the carbon alkyl chain in DTPP, did not affect mitochondrial morphology or energization state (scale = 10 *μ*m).

Since SS‐31 did not cause the same mitochondrial swelling as MitoQ, even at a very high dose, we considered that the toxic effects observed with the latter are probably unrelated to its anti‐oxidant activity. To investigate this, we assessed the individual effects of different structural components of MitoQ. We found that TPP (500 nmol/L), the mitochondrial targeting cation of MitoQ, did not induce any acute deleterious effects on mitochondria in OK cells (Fig. 2A–B). However, dodecyltriphenylphosphomium (DTPP) (500 nmol/L), which consists of TPP plus the carbon alkyl chain (but lacks the anti‐oxidant quinone), caused acute mitochondrial swelling and depolarization, identical to that induced by MitoQ (Fig. 2A–B). These findings suggest that the toxic effect of MitoQ on PT cell mitochondria is indeed unrelated to anti‐oxidant activity, and that the carbon alkyl chain plays an important role.

### MitoQ induces mitochondrial damage in kidney cortex tissue

**Figure 3:**
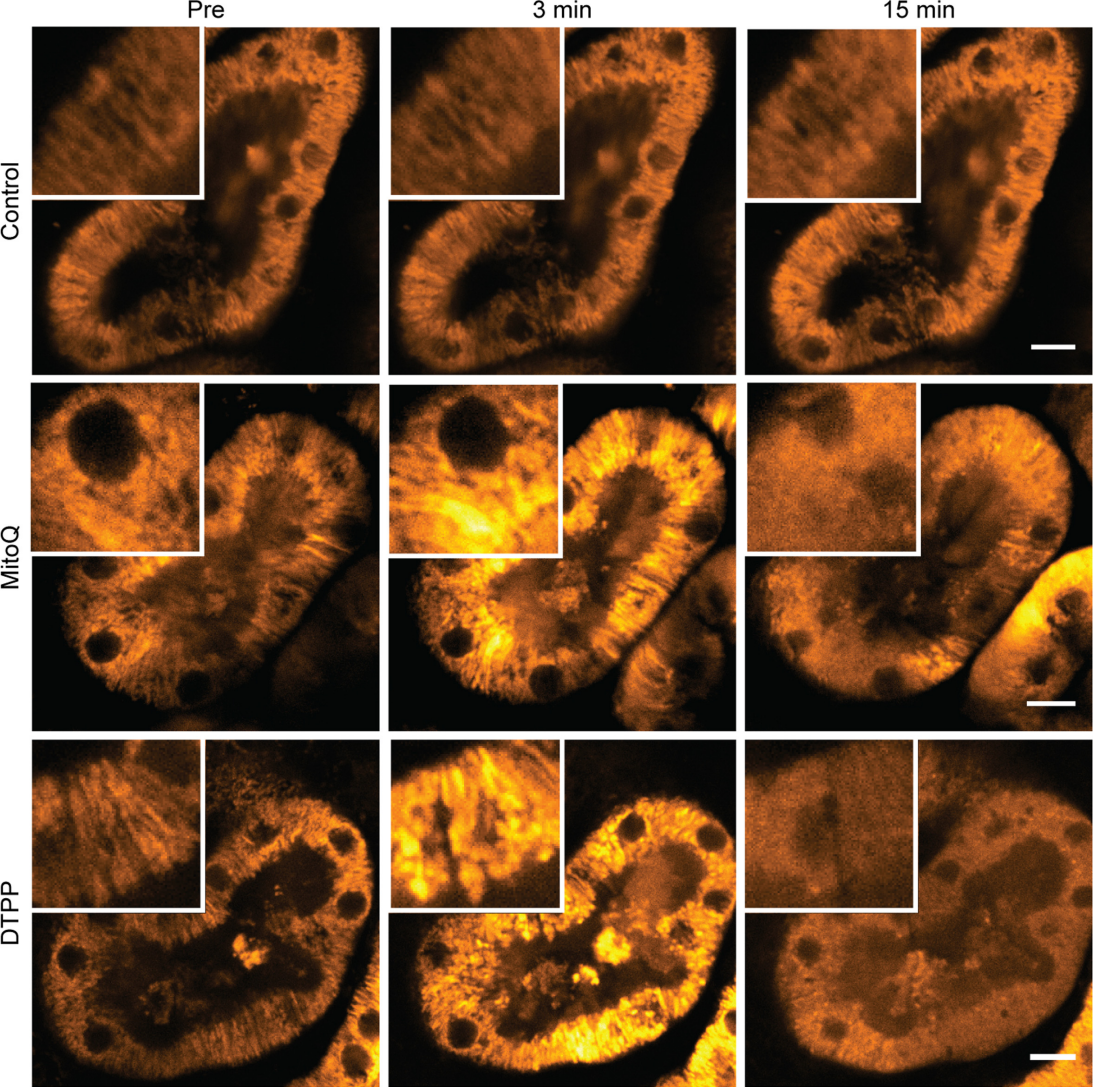
MitoQ induces mitochondrial swelling in kidney tissue. Live high‐resolution imaging with multiphoton microscopy of mouse kidney cortex sections loaded with TMRM revealed that MitoQ (50 *μ*mol/L) and DTPP (50 *μ*mol/L) both induced acute swelling of mitochondria in proximal tubules, followed subsequently by depolarization, demonstrated by redistribution of TMRM signal from mitochondria to the cytosol. In contrast, no such adverse changes were observed in mitochondria in control experiments over the same time period (scale = 10 *μ*m).

PT‐derived cell lines are recognized to have limitations as experimental models, and to be metabolically quite different from PT cells in vivo (Hall et al. 2010; Nieskens and Wilmer 2016). We therefore sought to know whether MitoQ also induces a similar phenotype in native PTs. To address this, we performed live imaging of freshly cut 250 *μ*m sections of mouse kidney cortex, using multiphoton microscopy and protocols that we established in previous studies (Hall et al. 2009). MitoQ (50 *μ*mol/L) was applied directly to the tissue, under tightly controlled experimental conditions. As in the OK cells, in response to MitoQ we observed rapid swelling and depolarization of mitochondria in PTs (Fig. 3). In contrast, over the same time period mitochondria in control tissue remained energized (Fig. 3). The effects of MitoQ were recreated by the addition of DTPP (50 *μ*mol/L) (Fig. 3). Thus, we confirmed that MitoQ causes swelling and depolarization of mitochondria in native PTs, and that this effect is due to the major structural backbone of the molecule, rather than the anti‐oxidant quinone component.

### Dodecyltriphenylphosphomium increases mitochondrial inner membrane permeability

**Figure 4:**
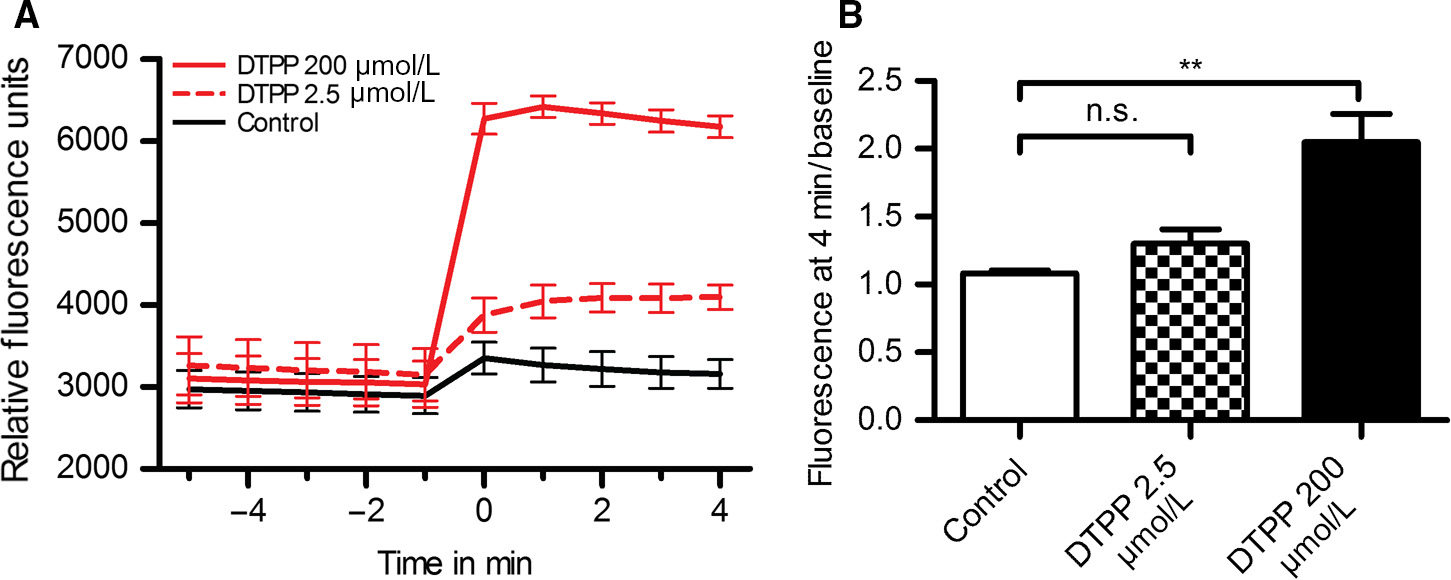
DTPP increases permeability in lipid vesicles resembling the mitochondrial inner membrane. (A) DTPP increased Calcein leakage from artificial liposomes (500 ng/mL) with a cardiolipin content of 20%, resembling the inner mitochondrial membrane. (B) Calcein fluorescence values at 4 min postdrug addition, normalized to baseline, one‐way ANOVA, post hoc Dunnett's multiple comparison test (** = *P* < 0.01, *n* = 3).

We hypothesized that mitochondrial swelling and depolarization induced by DTPP could be explained by an increase in IMM permeability, possibly due to insertion of the carbon alkyl chain. To investigate this, and exclude the potentially confounding effects of interactions with native mitochondrial proteins, we performed experiments using artificial liposomes containing 20% cardiolipin, to model the consistency of the IMM. These were filled with the fluorescent dye Calcein, release and unquenching of which was then used as a readout of increase in membrane permeability (Firsov et al. 2016). We observed an abrupt increase in Calcein release with DTPP (Fig. 4), suggesting that this compound can indeed directly increase permeability of a membrane closely resembling the IMM.

## Discussion

The realization that mitochondrial dysfunction is central to the pathogenesis of many kidney diseases, and that excess ROS generation within mitochondria can cause oxidative stress, has led to an intensive search for novel therapies that can lower ROS production specifically within these organelles. MitoQ is an anti‐oxidant that accumulates selectively into mitochondria at very high concentration, including in the kidney in vivo, and numerous previous studies have suggested that it might have beneficial effects in preclinical models of various different renal diseases. However, using live imaging and both in vitro and ex vivo models, we have now shown that MitoQ causes acute mitochondrial swelling and depolarization in PT cells, and that this effect is due to increased IMM permeability, rather than anti‐oxidant activity. These findings suggest that caution should be exercised before using this compound in renal patients.

Several different mitochondrial targeted anti‐oxidants have recently been developed that have generated much excitement and stimulated ongoing trials in humans. Whilst it seems clear that experimentally all of these agents can lower ROS levels in mitochondria and prevent oxidative stress, rather less attention has been paid to potential off‐target effects, which might be unique to each compound. However, evidence is emerging that drug specific effects on mitochondria can indeed occur. For example, it has recently been reported that the protective effects of SS peptides might be due in part to a favorable interaction with cardiolipin and cytochrome c (Birk et al. 2014). Given the generally dismal translational record of putative new kidney treatments, and the occurrence of unexpected toxic effects with previous attempts to pharmacologically prevent oxidative stress (e.g., bardoxolone in diabetic nephropathy (Van et al. 2015)), it is important to understand in as much detail as possible exactly what new drugs do to cell and organelle function in the kidney, before moving to clinical studies.

We have made the surprising and unexpected finding that MitoQ causes acute mitochondrial swelling and depolarization in PT cells. Since this effect can be recreated by DTPP, which lacks the quinone group of MitoQ, but not by SS‐31, it is highly unlikely to be related to anti‐oxidant activity. Moreover, we did not observe a decrease in OCR, suggesting that OXPHOS function is not inhibited. Acute mitochondrial swelling in pathological states is often attributed to the opening of a large pore in the IMM called the mitochondrial permeability transition pore (mPTP) (Biasutto et al. 2016). However, since we showed that DTPP increased the permeability of liposomes, which lack the components of the mPTP, a direct effect on membrane properties is a more likely explanation for mitochondrial swelling, which is biologically plausible as the alkyl chain is thought to insert into the IMM. Although some recent reports have raised concerns about adverse effects of MitoQ, to the best of our knowledge our study is the first to directly demonstrate rapid mitochondrial swelling and dissipation of Δ*ψ*_m_ in mammalian cells, and to provide a clear mechanistic explanation for this effect. Of note, a previous group reported evidence of mitochondrial swelling induced by DTPP in isolated yeast mitochondria (Trendeleva et al. 2012).

In striking contrast to scant reports of toxicity, numerous studies have reported beneficial effects for MitoQ, and we believe there are several potential explanations for this apparent discrepancy. First, many studies have relied on methodologies other than live imaging, and have naturally focused predominantly on alterations in ROS levels, so may have missed off‐target effects on mitochondrial morphology and energization. Remarkably, despite the fact that MitoQ causes severe mitochondrial swelling, OCR measurements suggested that OXPHOS can continue under these conditions. Importantly, this means that MitoQ and DTPP would not be identified as mitochondrial toxins if relying on this frequently used readout alone, and nicely demonstrates that live imaging can add substantial information to standard biochemical assays. Second, it could be that the concentration of MitoQ in renal mitochondria in vivo may not reach a sufficiently high level to cause toxicity. We are not aware of any previous kidney studies that have reported blood concentrations of MitoQ to directly relate our data to, but we found clear evidence of deleterious effects in vitro at a concentration of only 500 nmol/L. Moreover, evidence suggests that MitoQ does not undergo significant modification or metabolism in vivo (Smith and Murphy 2010). Third, there could be some beneficial effects of increasing IMM permeability in cells in disease states. For example, since ROS production is thought to be dependent on energization state (Murphy 2009), lowering Δ*ψ*_m_ might decrease ROS generation (so‐called partial uncoupling (Johnson‐Cadwell et al. 2007)). However, this has to be balanced with the risk of triggering other Δ*ψ*_m_‐dependent processes, like mitophagy (Kim and Lemasters 2011). Finally, the well‐recognized issue of publication bias is likely to play a role, as there is generally far more interest in reporting new treatments than new toxins.

In summary, we have found that MitoQ can cause significant mitochondrial toxicity in kidney tissue independent of anti‐oxidant activity, and in the absence of a decrease in OCR. These surprising findings underline the point that drugs can have unexpected side‐effects independent of their intended mechanism of action, and provide further evidence that live imaging can play an important role in comprehensively phenotyping how mitochondria respond to pharmacological interventions.

## Conflict of Interest

All the authors declared no competing interests.
